# Teachers' Perceptions of Applying Contemporary Skill Acquisition Approaches in High School Physical Education

**DOI:** 10.3389/fspor.2021.775423

**Published:** 2021-12-13

**Authors:** Sarah-Kate Millar, Jia Yi Chow, Mo Gleeson, Michelle Cleaver

**Affiliations:** ^1^Department of Health Sciences, University of Canterbury, Christchurch, New Zealand; ^2^Auckland University of Technology, Auckland, New Zealand; ^3^National Institute of Education, Nanyang Technological University, Singapore, Singapore; ^4^Long Bay College, Auckland, New Zealand; ^5^Glenfield College, Auckland, New Zealand

**Keywords:** acculturation, profession development, pedagogical practice, constraints-led approach (CLA), CLA

## Abstract

Using contemporary skill acquisition approaches to skill learning appears to be a worthwhile pedagogical option for teachers and coaches in sports and Physical Education (PE). However, PE at the High School level in New Zealand has assessment components that are still underpinned by traditional and outdated skill learning theories. In response to this challenge, two motivated Heads of Department in PE undertook a department-wide professional development initiative to teach the national standard assessment *via* the use of a contemporary skill acquisition approach, which is student-centred, with an emphasis on enhancing exploratory learning and encouraging autonomy. Each department worked together over a 10-week period with a Higher Education specialist in skill acquisition to design and teach using contemporary skill acquisition approaches. Qualitative data was collected *via* semi-structured focus group interviews. Insightful data on the influence of teaching using contemporary skill acquisition approaches was acquired from the teachers in the two PE departments. It was found that substantial pedagogical practice changes were achieved by the teachers (e.g., less focus on ideal technique and more on varying the context). They also enjoyed the learning experience that the contemporary skill acquisition approach offered as compared to their previous experience of more traditional teaching approaches, which have a focus on knowledge acquirement with little opportunities for exploratory learning. In addition, from a practical perspective, teachers were observed to demonstrate greater engagement in professional conversations around learning and could see greater relevance in the transfer of learning in the use of contemporary skill acquisition approaches to other teaching contexts.

## Introduction

Following international trends in using innovative and contemporary approaches in skill acquisition (e.g., Richard et al., 2018; Roberts et al., 2018; Orth et al., 2019), New Zealand Physical Education (PE) teachers are likewise, excited with the possible potential that such approaches could offer in enhancing pedagogical practices. In particular, the work by Chow (2013), Renshaw et al. (2015), and Moy et al. (2016) challenge the perceived relevance of more traditional approaches in teaching movement skills and purporting the advantage that contemporary approaches bring, in terms of enhancing motivation to learn. The article by Renshaw et al. (2015) was in our view, particularly insightful for teachers, who were accustomed to teaching using Play Practice, Games Concept Approaches (GCA) as well as Teaching Games for Understanding (TGfU) (see Bunker and Thorpe, 1982). Contemporary teaching methods like a Constraints-led approach (CLA) to skill acquisition (Newell, 1986; Davids et al., 2008) are gaining traction among practitioners (see Button et al., 2020). This current study outlines the response to the call from Renshaw and Chow (2019) for the need to undertake further research in authentic settings that are realistic in terms of the time needed to design learning activities. Importantly, we want to examine the impact of contemporary skill acquisition approaches in potentially enhancing pedagogical practices for PE teachers.

Notably, how skills are developed can be viewed predominately from two perspectives and they are namely, a traditional view or a contemporary approach. A traditional view is skills focused, with an emphasis on repetition to develop an optimal consistent movement pattern across individuals. From a traditional skill acquisition perspective, there is a specific and expected way a skill is to be performed, framed by a mental picture of this skill or movement that is to be learned. Importantly, changes to this mental picture are directed *via* internal changes (Renshaw and Chow, 2019). Teaching using this traditional view is characterized by the breaking down of skills into parts and then added back together with an emphasis on consistency in the expected movement form (i.e., a focus on task decomposition). An example of this could be TGfU (Bunker and Thorpe, 1982), where games are used to highlight predetermined game concepts. These traditional assumptions are clear, with an overall aim for the learners to explain their performance often *via* the use of questioning (Chow et al., 2016).

Contemporary skill acquisition approaches in this paper relates to a Constraints-led approach (CLA) that is underpinned by ecological dynamics. Learners are provided with opportunities to explore various individualized movement solutions where the goal-directed behavior emerges or is self-organized as a consequence of the interactions among the constraints (i.e., task, environment, and performer) present in the teaching and learning context. Driven from an ecological dynamics framework, CLA focuses on the learning for the individual, as opposed to viewing it as a specific teaching strategy (Renshaw et al., 2015). An essential principle of a CLA is the mutual relationship(s) that emerges between the individual(s) and their environment (Renshaw and Chow, 2019). Coordination that emerges, and is acquired by learners, is a consequence of the continued interaction between the individual and environment (Button et al., 2020). Despite the advances in the body of knowledge in skill acquisition, and the interaction between the individual and environment, the use of traditional theoretical approaches like Fitts and Posner (Fitts, 1964; Fitts and Posner, 1967) stages of learning to understand skill acquisition remains prevalent in schools today (Moy et al., 2016).

An investigation using the Physical Education New Zealand (PENZ) teacher network in Auckland reported that although schools were employing new graduate teachers who had experience in contemporary movement approaches taught at University, traditional methods are still widely used in New Zealand schools (M. Clever, personal communication, June 6th, 2016). More critically, when these new graduate teachers were confronted with school programmes, they found existing teachers and students still using traditional movement approaches of motor skill learning in their practice. This acculturation of teaching practice is heavily influenced by how they were taught when they were at school, and how they see their senior colleagues teaching. This has clear implications on the importance of teachers having a deeper understanding and application of contemporary skill acquisition theories (Moy et al., 2014).

Further insights were garnered when two Heads of Department (from two large Auckland Schools) begun working with AUT University to examine the key factor in the acculturation of teaching practice. Specifically, it was found that the current nation-wide Achievement Standard (AS) or unit(s) of National assessed work required for students to complete in High School Physical Education were using traditional movement theories of motor skill learning in their explanation of what was required for a certain AS (i.e., AS 90967, which specifically focused on *knowledge of technique and quality of practice*) (“NZQA” n.d.). New Zealand has a qualification system (Achievement Standards for Secondary Education in Physical Education) and this requires students to complete a number of AS units in order for them to attain their national qualification (AS 90967 is one of these required AS unit). To compound the challenge for teachers further, the exemplars of excellence and the marking schedule are pegged to traditional theories (e.g., stages of learning by Fitts and Posner, 1967). Therefore, there were two large impacting factors influencing the teaching practices employed in schools; an acculturation effect of new(er) teachers observing senior teachers practice using traditional theory, as well as the nature of the High School Physical Education AS assessments promoting the use of this outdated traditional motor-learning theories. The importance of structure and content of national achievement standards directly influenced not only the teachers' choice of content (in this case traditional theories), but also their teaching styles. A study by Desimone and colleagues in 2009 found that authority directly influences teachers' behavior and in particular, their instructional strategies and interactions with other teachers and students. An example of authority in New Zealand would be the structure, suggested assessment examples and language used to pass a particular National Achievement Standard.

Moy et al. (2016) explored the challenge of using alternative contemporary skill acquisition pedagogies to those traditionally used in schools with a small group of student teacher participants. In particular, they compared the use of a CLA to traditional games based approaches like TGfU, and examined the teaching experiences of the participants; which found that teachers felt it was more inclusive to students and more effective in skill development. This current study focuses on investigating an existing achievement standard (an external assessed unit of work) as the context to explore the change. That is, we wanted to know what the teachers' experience would be in moving away from teaching traditional motor skill theory to teaching contemporary skill acquisition approaches. In addition, in order to examine a strategy to try and help alleviate the acculturation effect of new teachers predominately observing teaching methods of how they used to be taught; this study included a professional development approach with the Head of Department, and their teachers to work together to upskill on contemporary skill acquisition knowledge and approaches to teach a new assessment/unit of work together.

The role of professional development (PD) for teachers has several overarching aims, but primarily, it is to have a positive impact on students. A model by Desimone (2009), suggests that successful PD can result in a change in teacher attitudes and beliefs, as well as their teaching, which subsequently has an impact of students' learning. The positive relationship between teacher change through PD and student achievement has been well-documented (e.g., Guskey and Sparks, 1996; Hattie, 2008), as teachers are considered the most important factor in students' achievement. There are various models of teacher PD; from short time frame intensive blocks to others, which cover a much longer period of time. The benefits of PD over a longer time period include the opportunities for interactive and non-recursive relationships to be established (Desimone, 2009), as well as time for deeper thinking on a topic. In addition to the length of time of the PD, the context is also shown to have an impact on teaching practice. In particular, the use of an authentic context to learn and explore new knowledge, has a stronger impact than an artificial context (Guskey, 2002; Sofo and Curtner-Smith, 2010).

The aim of this study is to explore the teaching and learning experiences of a group of teachers and two HODs, as they taught an existing AS or unit of work using contemporary skill acquisition knowledge, instead of using traditional motor skill theory. The teaching of this AS, which occurred over a pro-longed time period (10 weeks), was a focused PD activity for each school. The teachers were required to up-skill their knowledge of contemporary skill acquisition approaches to teach their students.

## Materials and Methods

### Participants and Setting

Teaching teams for two large High Schools in Auckland volunteered to be involved in this project. Their participation came as a result of each schools highly motivated HOD interest in the topic area and project and would be considered a convenient sample (Patton, 2015). Both HOD's had been involved in informally upskilling themselves in contemporary skill acquisition knowledge over the preceding year in order to help teach a different group of Senior Physical Education students. School 1 involved 4 teachers, the HOD with 23 years teaching experience and 3 junior teachers with between 2 and 3 years of experience each. School 2 involved 3 teachers, the HOD with 18 years teaching experience, while the other two teachers were one with 17 years' experience and the other, a junior teacher with 3 years of teaching experience. The other key participant in this study was a University or Higher Education (HE) researcher and lecturer. This person was involved as a skill acquisition specialist, who has 7 years of experience in High School teaching, prior to 12 years of University teaching and research in contemporary skill acquisition approaches. The HE lecturer not only knew the theory and the High School PE teaching environment, but had worked with the two HOD's before in a professional development setting. University ethics was obtained and all participants consented to be involved in the study voluntarily, and all names used in this paper are pseudonyms to ensure their anonymity.

### Study Design

**Figure 1:**
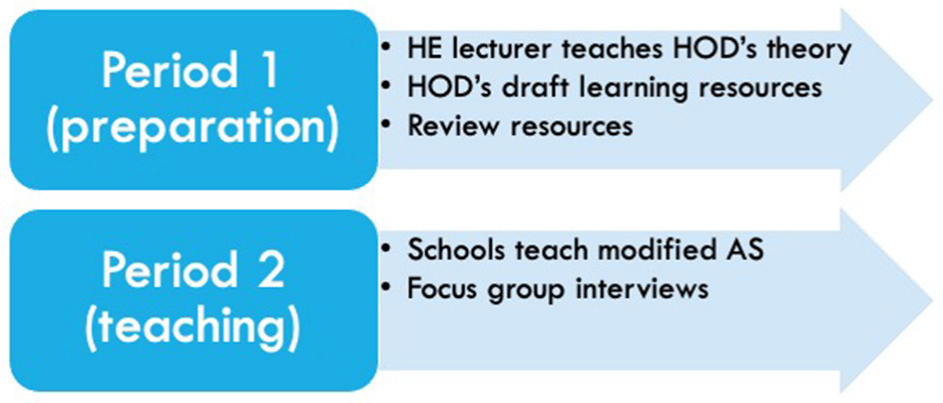
Schematic of study design two periods.

The duration of the project was 9 months; see Figure 1 and this was divided into two distinct time periods; a preparation period and a teaching period. The first period only involved the two HOD's and the HE lecturer, whereas the second period involved the two schools teaching the modified AS over a 10 week time window alongside the HOD.

Based on the HOD's previous learning of contemporary skill acquisition and where they felt the biggest benefits were for students of these approaches, it was decided that in order to best teach the foundations of contemporary skill acquisition approaches for teaching the modified AS, the content needed to be broken down into six topics. The six topics^1^ that are the key basis for any beginner to have a foundational understanding of contemporary approaches were: (1) Learning, (2) Perception drives Action, (3) The Constraints-led approach, (4) Newell's model of Skill Acquisition, (5) Learning compared to Performance, and (6) Representative learning design.

#### Period One (Months 1–6)

Period one, preparation was months 1–6, and involved 6 weekly 1 h teaching sessions by the HE lecturer on each topic, followed by 1 h of brainstorming of different teaching activities for teachers and students on the topic. At the conclusion of the 6 weeks of theory teaching and brainstorming, the two HOD's wrote teaching and learning activities for the six topics. This writing process occurred over an 8 week time window and concluded in a peer review process with the HE lecturer on the choice of teaching and learning activities, as well as checking for accuracy of contemporary skill acquisition knowledge. The designing of teaching and learning activities also involved the collection of supporting resources that all the teachers could use to teach the modified AS.

#### Period Two (Months 7–9)

Period two, teaching (and reflection) was during months 7–9 and involved the two schools which was led by the HOD's teaching the modified AS based on contemporary approaches over a 10 week time period. In addition to teaching the AS *via* the planned lessons, each school chose the context for the assessment of the AS. School 1 chose handball and School 2 selected football. Focus group interviews were conducted for the teachers at the end of the 10 week time period in month 9, to explore the teaching and learning experiences of the modified AS by both schools.

### Data Collection

To address the aim of this study, two semi-structured focus group interviews were conducted at the conclusion of teaching the 10 week AS; one with each school. Each interview lasted between 45 and 60 mins and were conducted by the HE lecturer who previously taught High School Physical Education and thus has an understanding of the teaching environment, as well as intimate knowledge of the modified AS. The two interviews allowed for a degree of conversation and discussion to develop around the general questions. There are advantages of the interviewer being involved in the research design of the AS (Millward, 1995), and who is also familiar with the participants (McLafferty, 2004). According to Bonner and Tolhurst (2002), having an interviewer who has a wealth of knowledge and expertise in the area, a superior understanding of the AS design and the ability to naturally interact with the participants, allows for more insightful understanding of the teachers' responses.

Specifically, the focus group interviews targeted the following question areas in order to best understand the effect of teaching the modified AS and also potential changes that could improve it; (1) teachers' experiences, (2) advantages/disadvantages for teachers, (3) influence of teaching modified AS on other teaching practices, and (4) recommendations for further modifications. Interviews from the two schools were audio recorded and then electronically transcribed verbatim by the researcher.

### Data Analysis

Focus group data was rigorously transcribed verbatim from securely saved audio recordings. A thematic analysis was used, and this was based on Braun and Clarke's (2006) guidelines and used an inductive approach, where analysis is ultimately driven by the data. The six stages of Braun and Clarke's (2006) thematic analysis were; (1) Primary researcher familiarizing self with data, (2) generating initial codes, (3) searching for themes, (4) reviewing themes, (5) defining themes, and (6) producing a report. Importantly, this inductive method allowed participants to talk about their own experiences in their own words and to elaborate where necessary (Gratton and Jones, 2004). Higher and lower order themes emerged from the thematic data analysis process.

## Results

**Figure 2:**
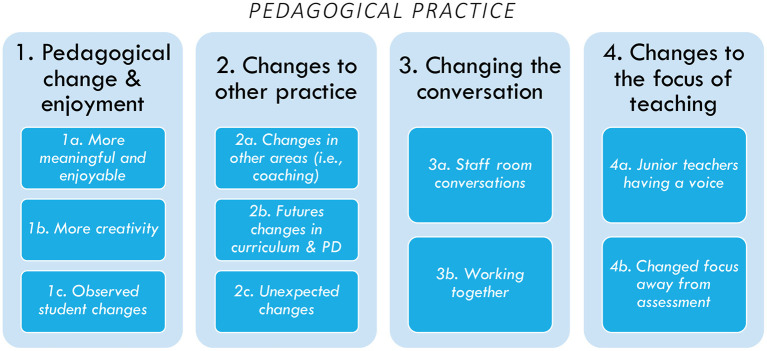
Pedagogical practice 4 higher order themes and subsequent 2–3 lower order themes.

From the focus group interviews with all participants, an overarching main theme of “pedagogical practices” emerged, in addition to four higher order themes and a number of inductive lower order themes; see Figure 2 below. The higher order themes were: (1) Pedagogical change and enjoyment, (2) Changes to other practices, (3) Changing the conversation, and (4) Changes to the focus of teaching.

### Main Theme: Pedagogical Practices

Under the overarching theme of pedagogical practice, are the four higher order themes. In this paper, Pedagogical Practices would cover areas relating to how teachers plan, deliver and assess their own teaching. It would typically highlight the teaching practices and processes that the teacher would adopt to enact the lesson.

#### Pedagogical Change and Enjoyment

The first high order theme to emerge from focus group interviews with teachers from the two schools was the pedagogical change they noticed in themselves and the enjoyment the teachers found teaching using a contemporary approach.

##### More Engagement and Meaningful

From the start of focus group interviews, it became clear from not only the participants' conversations, but also their body language, that they clearly enjoyed teaching the modified AS. In particular, they enjoyed the change in focus to a contemporary approach, which they found to be more meaningful for the students' learning.

*I think the lessons are more meaningful definitely, like the old way, like I think the theory aspects of the old way was easier for them to get but the learning was more meaningful in the new way and just the tasks we did (S1, T3)*

*Just hearing these guys use different terminology and just actually them having the confidence to try different things and go, I kind of get what I am talking about and this is how I can now modify an activity to suit the kids and to change one of the constraints was so much better. (S2, T1)*

##### More Creativity

With the introduction of contemporary approaches, and in particular an understanding and appreciation that there are multiple movement solutions to a problem; the teachers were more creative with their teaching approaches than they had previously experienced with traditional motor learning theories.

*It was quite cool having, you know, when we did the practical lessons, using things that were nothing to do with the assessment (S1, T2)*

*We used three different balls, myself and (teacher) did an experiment with different balls at the same time. It was brilliant. (S2, T3)*

##### Observed Student Changes

While the focus of this study was not on the students and no data was collected from the students, the teachers could not help but talk about observed changes in the students. In particular, the teachers acknowledged the changes that were observed in the students' ability to make meaning from the content and how they could more easily transfer it to other contexts.

*I think it's way more enjoyable to have this fashion of learning anyway. To learn how to put it into place and for the students to learn what works and what doesn't for them (S2, T2)*

*I liked the change this year, that it is different that they are learning on their own to a point, when they are actually having to show something, act it and then write about it, so I like the change (S1, T4)*

#### Changes to Other Practice

The second higher order theme to emerge from the teachers' focus group interviews was how they noticed changes to their teaching in other classes (not the AS class) and/or changes in the way they coached their sports teams. In this respect, the pedagogical change is related to the change in teachers' philosophy. They also made quite strong mention of future changes they were already starting to think about with other classes and/or curriculum changes they thought would help students.

##### Changes in Other Areas (i.e., Coaching)

An important recurring theme from all participants interviewed was their enthusiasm about what they had learnt and how this could be transferred into other context for them. In particular, the participants also shared how they, as coaches in their own sports, have adopted a new contemporary approach.

*I coach basketball, volleyball, touch and netball. Yeah, I definitely like thought about including like some constraints into the training and making it more on the students like in terms for their successes (S1 T4)*

*I actually did, I used some of those activities which I use in my little 7 and 8 year old footballers, for my guys and it actually worked a treat (S2, T3)*

##### Futures Changes in Curriculum and PD

The teachers also started to recognize the need for change to happen in other parts of the curriculum and/or in the department.

*I actually think there's quite a lot of good learning that we did with this level that we should sit down with the senior units and maybe look at that may need some bumping up in level of knowledge now (S1, T1)*

*I think even having these kind of conversations there are so many opportunities for us to develop it further. But I think we'd definitely look at trying it, the same again next year and we've already talked about the next level up, changing that unit too, so that it actually meets, well it's using the same terminology (S2, T1)*

The teachers were also excited and motivated by the idea of how they have or will also use this new knowledge or approach with other classes as well as further professional development for the whole department.

*I am so different with my year 9s now (junior class), just the way you set things up and just kind of giving them different choices and getting them to make decisions as well which has been so different, you know, you just, it's not about teaching them the skill, it's about them. I found I am trying to get them to work out what works for them, and that's probably been my kind of thing. (S2, T1)*

*I think it could be really cool PD sessions for all our department to maybe just start using in year 9 and here's some concepts and here's how you might do it, because everyone's really used to using TGFU because we have done for years, so just starting to look at the difference and pros and cons for both and how you could give it a go. (S1, T1)*

##### Unexpected Changes

The teachers also observed unexpected behaviors that the students demonstrated while teaching the modified AS.

*They're like “oh my gosh”, like we actually learnt all this stuff when we were a baby and no-one taught us to do that, we learnt through observation or variable practise or experimentation (S1, T4)*

*We had the constraints lessons that we ran and we had like our goal and then like my kids came up with a completely different one or completely opposite to what I was getting (S1, T3)*

#### Changing the Conversation

The third major theme to emerge, which was quite unexpected from the outset of the study design, was the inter-personal benefits of not only learning and teaching using a contemporary approach, but also working together on something new at the same time.

##### Staff Room Conversations

A surprising observation that the teachers spoke enthusiastically about was how they noticed a change in the staff room conversations. In particular, there were more conversations about teaching and learning as compared to outside school conversations.

*We definitely had more conversations,……., we would all sit together so we were just all the time chatting about it aye. (S2, T3) Which was a nice conversation because we don't often have that about anything (S2, T1). Yeah, we normally talk about football (S2, T3)*

##### Working Together

The teachers spoke favorably of new experiences that teaching this modified AS brought to them. In particular, the joy of working together and the support they received by having a knowledgeable and approachable HOD was significant.

*It was really nice (HOD name) sat us down like each topic and we went through and we were able to ask questions or like prior into the lesson if I wasn't sure or anything I felt like I could ask (junior teacher name) or (HOD name) and see where I was at and make sure that I was delivering the correct content (S1, T4)*

*I thought it was good, it was really nice to have someone to talk to and then like, for example, like I say, like I might have been in a few lessons behind so therefore someone else had already done it so I was like “oh, can I ask what you did? Oh, that's a cool idea, I'm going to take that into my lesson next”. (S1, T4)*

*It was so helpful listening to what each other were doing, we could like steal ideas. (S1, T2)*

#### Changes to the Focus of Teaching

The fourth and final theme to emerge from the focus group interviews was the teachers' perceptions of how the focus of their teaching has dramatically shifted from an assessment focus to a teaching focus. In addition, the junior teachers in the departments felt they have more to contribute and could draw on previous knowledge they had.

##### Junior Teachers Having a Voice

*I will go through it the week before and, yeah, a lot of it was refreshing or it would be like, oh, okay, I don't remember any of that, so I had to pick it up a bit more but, yeah, like those, it was just really easy to pick up and just, yeah, just take on board really, and, yeah, it was a bit of a refresher and oh, I remember going through that now, and like you say, it was then looking at that and going, “yeah, I can do this” without even realising it so that when you then do it, put it into place, you are like, okay, this is how I definitely do it, and then, (S2, T3)*

*Yeah. I remember some of it like from uni and some of the papers but its like remembering it and then actually being able to deliver it, it is quite different. (S1, T4)*

##### Changed Focus Away From Assessment

A significant outcome from the teachers implementing this modified AS was the perceived change in their role in the classroom. In particular, there was a strong sense that they were “actually” teaching and the joy that they experienced.

*I thought like we're not teaching to the assessment which is I thought really nice because in hindsight every single assessment probably throughout the school you are teaching to the assessment (S1, T2)*

*We are employed to be educators not being people how to pass an assessment and we get so caught up in assessments that we don't teach the kids and so if we make sure the way that we introduce the assessment is actually, you've learnt this and now we are going to assess it, rather than this is what the assessment is. We actually need to teach the content and then worry about the assessment. (S2, T1)*

## Discussion

The aim of this study was to examine the teaching and learning experiences of teachers who taught a unit of work using a contemporary skill acquisition approach. Specifically, the study attempted to examine the teachers' perceived experiences of teaching a modified National Achievement Standard (AS) over a 10 week period. The teaching of this modified AS required the teachers to learn new contemporary skill acquisition approaches, and then teach and assess this knowledge. It was found in this study that the teaching and assessing of a modified AS in this study has allowed the teachers to not only learn contemporary skill acquisition approaches, but also make significant changes to their pedagogical practices. The latter cannot be underestimated, as this can be a challenging (unexpected) outcome to achieve, but as shown in this study, it has many benefits for the teachers (and their current and future students) since pedagogical practices are the pillars for teachers in planning, enacting and assessing their teaching. Below, we highlight some key discussion points related to the impact of using such approaches on the perceived enjoyment by the teachers, changes to pedagogical practices and other reported changes.

### Enjoyment and Boarder Pedagogical Changes

One key finding from this study relates to the greater enjoyment that the teachers reported in using contemporary skill acquisition approaches to teach the modified AS. The key theoretical framework relevant to the discussion of the findings from this study is the Self-Determination Theory (SDT) that emphasizes intrinsic motivation and how it is underpinned by an individual's basic psychological need to have a sense of autonomy, competency and relatedness (Deci and Ryan, 2000). Model of PD (Guskey, 1986, 2002) shows that a strong change occurred when the teachers' beliefs matched the observed behaviors. In this situation, they could not only see the students enjoy the content that was taught, but could also observe the success they were experiencing.

Teachers found that moving away from a traditional approach with ideal movements or expected outcomes allowed them to teach using a wide(r) range of contexts (e.g., skate boarding and different sports) and provided greater autonomy for the students to choose their own skills to develop. The advantages experienced here by these teachers' learning and teaching *via* contemporary approaches supports findings by Moy et al. (2016) who reported an increase in students' intrinsic motivation and enjoyment when teaching *via* a contemporary approach such as CLA compared to traditional approaches. There is indeed potential for such contemporary approaches that encourages exploratory learning to support stronger sense of autonomy and enjoyment (Chow et al., 2016; Rudd et al., 2021). The teachers also commented on the enjoyment the students had with contemporary approaches and the opportunity to explore movement in a different contexts. This finding is also similar to that of Moy et al. (2014) who observed participants experiencing enjoyment and understanding of the CLA framework. Although no data was collected from the students, an observation from the teachers was the enjoyment that the students experienced, and this could stem from the increased level of meaningfulness from their lessons. This is pertinent to how the students have a greater sense of autonomy in their learning as well as relatedness from a SDT perspective.

From the teachers' point of view, there was the realization of how much they enjoyed teaching *via* contemporary approaches. The sharing by teachers in this study helped to affirm how they were eager to try new ideas after their experience with these approaches. This is aligned to the recent findings by Lee et al. (2017) on how teachers were encouraged to create more representative learning designs that situate skills within more game-like settings once there were more familiar with contemporary skill acquisition approaches.

### Change to Other Practices

Another key finding from the current study is how teachers see the possibility of transferring the learning experiences to other contexts. This is especially relevant to how teachers may take ownership of their own learning and development. The sense of autonomy in the teachers can be enhanced as they can then potentially see how their experience using contemporary approaches can be used for other lessons and learning platforms. Teachers can be further encouraged to design their own professional development journey and this is evident of the pursuit for teachers to be self-directed learners. This is non-trivial as there is a sensible push on the part of the students and teachers to move away from some status quo of a teacher-driven approach to teach and learn. As pointed by Moy et al. (2014), an alternative approach, such as the CLA, can be attractive when is it underpinned by learning theory that is operationalized in a research-informed pedagogical learning design which helps students perceive the effectiveness through experiencing or seeing that it works.

While not specifically measured in this study, the excitement that was observed among the teachers on new knowledge could indicate a heightened sense of competency that was afforded through the contemporary approaches experience. The point raised by the teachers on sharing teaching ideas with the rest of the department is very relevant to how the teachers acquire a sense of being part of the community and the possibility of playing a mentoring role with such new knowledge and passion.

A contributing factor to the success and enjoyment expressed by these teachers was the purposeful structure to the PD. In particular, the current study was undertaken over a relatively long period of time and such a feature has been attributed to successful PD outcomes (Desimone, 2009). Potentially, a greater factor on the success of this project, was the role the HOD played. The direct leadership involved in the PD plays a powerful role in teacher change (Leithwood et al., 2004; Marzano et al., 2007). Direct leadership incorporates staff developers, subject specialist and director coordinators like a Head of Department (HOD). Direct leaders (i.e., HOD) set the direction for PD, as well as influence the environment for natural change in practice, especially when they recognize how new practices can help students (Leithwood et al., 2004). It is also clear that when direct leaders work in collaboration with other teachers, there is a sharing of leadership, strategic thinking, as well as opportunities to learn off from each other (Coplan and Knapp, 2006). As a senior member of staff, they set the direction and can influence the environment (Marzano et al., 2007) and to have the HOD learning alongside the teachers contributed to an authentic PD learning setting was considered supportive for the teachers (Leithwood et al., 2004).

### Changed Staff Room Conversations -Working Together

From the interview data, it was also determined that teachers were able to engage in more meaningful conversations relating to the teaching experience of the modified AS based on contemporary approaches. The conversations were focused on teaching and learning rather than outside school conversations. This is a positive observation as it reinforces how teachers can consolidate and build on their teachers' identity with such discourse among colleagues in the school context (Yuan and Mak, 2018).

Mentoring of more junior colleagues by senior staff could also likely to occur with greater regularity when such conversations become more pervasive with the introduction of contemporary approaches that creates a renewed interest to enhance teaching and learning. With reference to teacher professionalism, this is one key professional value that would be strongly encouraged among educators to see themselves as a part of a larger professional community (Tan et al., 2017). The sharing of previous knowledge and experiences is an excellent channel for teachers to engage in professional conversations that can develop a stronger sense of professionalism among peers (Liu, 2021).

A reported barrier by Lawson (1986), and Moy et al. (2016) for why contemporary approaches may not have been employed earlier in the High School setting was an acculturation of teaching practice. This study has enabled the more junior staff with previous knowledge in contemporary skill acquisition approaches a chance to not only use this knowledge but also share this with their colleagues and therefore help be a valuable team member. The power for junior teachers to help overcome this acculturation effect has been reported in literature (i.e., Li and Cruz, 2008; Wang and Ha, 2012) and the similar effects can be observed in this study.

### Changed Focus of Teaching–Away From Assessment

Literature from Rink (2001) about the structure of lessons to be more integrative between theory and practice is relevant as students are challenged to be more explorative than their learning experiences with traditional lessons. This is important as such a platform would allow everyone the opportunity to find success and make meaning for themselves (Morgan and Hansen, 2008). This point relates to how there could be different pathways to achieve success of intended outcome. One key conceptual idea from an ecological dynamics perspective is relevant to the point on degeneracy and how a contemporary skill acquisition approach (e.g., Constraints-led Approach) can provide the platform for emergence of degenerate behaviors (Button et al., 2020). The concept of degeneracy accounts for how neurobiological systems have the capacity to achieve the same or different outcomes in varying situations, with structurally different components of the musculoskeletal subsystem (Edelman and Gally, 2001; Hong and Newell, 2006). Key questions that can be asked are aligned to how and if the assessment rubrics can be reviewed to account for the possibility of greater exploratory behaviors and thus learning. Assessment shapes behaviors and a change in the focus of teaching (i.e., away from summative assessment and to more formative assessment descriptors that can account for exploratory learning) would also be impacted by how assessment criteria are established (Chan et al., 2011; Tolgfors, 2018). For example, teachers would more likely to design learning activities that emphasizes exploratory learning and moving away from a form-focused set of instructions when the assessment rubrics describes learning outcomes that are aligned to adaptability (e.g., throwing to different directions and distances rather than the form of the throwing action).

### Conclusion and Implications for Policy and Practice

Results in this study suggest that teachers appreciated a change in their pedagogical practices. Evidence from the teachers also indicated the increased conversations present in the school staff room around pedagogy and inquiry into teaching practice. These informal conversations in the staffroom and reflections appear to have prompted changes to their own practice which have the potential to transfer into areas beyond the context of the modified AS. This transfer of teachers' knowledge demonstrates how research into one's own pedagogy can be synthesized across a range of teaching subjects, not just in physical education. Moving forward, it would indeed be valuable to examine students' data in such studies to gain additional perspective on the impact upon students' learning experience.

The findings have direct implications on how teacher education can be designed and delivered. For example, there could be concerted efforts to develop closer collaborations between schools and University to strengthen the theory-practice nexus pertaining to the use of such contemporary skill acquisition approaches to further support teaching and learning. Academics could be encouraged to work closer with practitioners to share new insights and advancement in theory but with a view to impact real learning in the school landscape. Practitioners may also feel more empowered to be part of such collaborations in strengthening the practice to enhance pedagogical practices to support more exploratory learning. Policy makers can also consider the impact of adopting such a pedagogical approach on professional development for existing teachers in the school especially with reference to acculturalisation. These revisions and enhancements could be captured in revised curriculum designs that can inform practitioners about the potential benefits of such approaches. Comprehensive PD programmes can then be designed in tandem with the revised curriculum to promote translation of these new knowledge and practices.

## Data Availability Statement

The raw data supporting the conclusions of this article will be made available by the authors, without undue reservation.

## Ethics Statement

The studies involving human participants were reviewed and approved by Auckland Universtiy of Technology Ethics Committee. The patients/participants provided their written informed consent to participate in this study.

## Author Contributions

S-KM co-designed the study with the MG and MC. S-KM did the analysis and wrote the initial research draft. JC lead the final write up. All authors contributed to the article and approved the submitted version.

^2^

## Conflict of Interest

The authors declare that the research was conducted in the absence of any commercial or financial relationships that could be construed as a potential conflict of interest.

## Publisher's Note

All claims expressed in this article are solely those of the authors and do not necessarily represent those of their affiliated organizations, or those of the publisher, the editors and the reviewers. Any product that may be evaluated in this article, or claim that may be made by its manufacturer, is not guaranteed or endorsed by the publisher.

## Footnotes

^1^Resource for Physical Education Teachers: https://www.aut.ac.nz/research/academic-departments/health-physical-education-and-coaching/pe-teaching-and-research.

^2^ https://www.aut.ac.nz/research/academic-departments/health-physical-education-and-coaching/pe-teaching-and-research
